# Impacts of crop rotational diversity and grazing under integrated crop-livestock system on soil surface greenhouse gas fluxes

**DOI:** 10.1371/journal.pone.0217069

**Published:** 2019-05-22

**Authors:** Gandura Omar Abagandura, Songul Şentürklü, Navdeep Singh, Sandeep Kumar, Douglas G. Landblom, Kris Ringwall

**Affiliations:** 1 Department of Agronomy, Horticulture and Plant Science, South Dakota State University, Brookings, South Dakota, United States of America; 2 Dickinson Research Extension Center, North Dakota State University, Dickinson, North Dakota, United States of America; 3 Animal Science Department, Canakkale Onsekiz Mart Universitesi, Canakkale, Turkey; Consiglio per la Ricerca e la Sperimentazione in Agricoltura, ITALY

## Abstract

Integrated crop-livestock (ICL) system is beneficial in enhancing soil organic carbon and nutrient cycling. However, the benefits of the ICL system on mitigation of GHG emissions are poorly understood. Thus, the present study was initiated in 2011 to assess the effect of crop rotation diversity and grazing managed under the ICL system on GHG emissions. The cropping system investigated here included spring wheat grown continuously for five years and a 5-yr crop rotation (spring wheat-cover crops-corn-pea/barley-sunflower). Each phase was present each year. Yearling steers grazed only the pea/barley, corn and cover crops plots in 2016 and 2017. Exclusion areas avoided the grazing in these crops to compare the GHG fluxes under grazed vs. non-grazed areas. The GHG fluxes were measured weekly from all crop phases during the growing season for both years using a static chamber. Cumulative CO_2_ and CH_4_ fluxes were similar from all crop phases over the study period. However, continuous spring wheat recorded higher cumulative N_2_O fluxes (671 g N ha^-1^) than that under spring wheat in rotation (571 g N ha^-1^). Grazing decreased cumulative CO_2_ fluxes (359 kg C ha^-1^) compared to ungrazed (409 kg C ha^-1)^, however, no effect from grazing on cumulative CH_4_ and N_2_O fluxes over the study period were found. The present study shows that grazing and crop rotational diversity affected carbon and nitrogen inputs, which in turn affected soil CO_2_ and N_2_O fluxes. Long-term monitoring is needed to evaluate the response of soil GHG emissions to grazing and crop rotation interactions under the ICL system.

## Introduction

Increasing atmospheric concentrations of greenhouse gases [GHG, particularly carbon dioxide (CO_2_), methane (CH_4_), and nitrous oxide (N_2_O)] are contributing to increasing trends in global warming [1]. Agriculture is one of the sources of worldwide GHG emissions, accounting for 9% of total U.S. GHG emissions [2]. Integrated crop-livestock (ICL) system can play a prominent role in GHG emissions mitigation [3–5]. Few examples of commonly implemented ICL system in the U.S. include: animal grazing of cover crops within cash crop rotations, crop residue grazing, silvopasture and agroforestry, sod based crop rotation (perennial forage for grazing with crops), dual purpose cereal crops (harvesting for grains followed by grazing e.g. corn) [6]. Crop rotation and grazing under the ICL system can impact soil organic carbon (SOC), crop residue mineralization, and root and microbial respiration [7–8], thus play a major role in regulating soil surface GHG emissions [9].

Crop residues returned to the soil are the main input in maintaining SOC, which generally seems to increase with the diversified crop rotation compared to the mono-cropping [10–11], which if employed continuously increases the need for off-farm inputs due to increased infestation of weeds, diseases, and pests [12–13]. Crop rotation affects numerous soil properties, including water holding capacity, nutrient availability, and soil structure [14–15, 9], all of those can influence soil GHG emissions. For example, crop type can impact soil temperature and water content by affecting shade intensity and evapotranspiration [16, 11].

There are few studies investigating the effect of crop rotational diversity on GHG emissions from different plant species within the rotation. Wegner et al. [17], who monitored GHG fluxes under corn and soybean rotation (both phases were present each year) for three years, found that corn and soybean emitted similar soil CH_4_ fluxes each year. In addition, Lehman et al. [18] reported no significant differences on N_2_O fluxes from different plant species (corn-pea-winter wheat-soybean, each phase was present every year) within the rotation for four years. However, Halvorson and Del Grosso [19] reported that CO_2_ emissions were influenced by plant species within the rotation in Northeastern Colorado, with barley (corn-barley rotation) emitting higher cumulative CO_2_ flux compared to corn (continuous corn) and dry beans (corn-dry beans rotation) in the same year. These differences on cumulative CO_2_ flux were attributed to the amount and quality of decomposing residue from the previous crops.

Other studies have compared soil GHG fluxes from a crop grown in rotation with a similar crop grown in mono-cropping, and their results were contradictory. For example, corn in rotation has shown to reduce total N_2_O as well as CO_2_ emissions compared with continuous corn [20–22] due to the increased fertilizer input from the mono-cropping. Sainju et al. [16] reported higher CH_4_ uptake by barley from a barley–pea rotation than from continuous barley. However, other studies have reported no effect on soil GHG from crop rotation compared to mono-cropping. According to Barton et al. [23], wheat in rotation (lupin–wheat) has been found to emit similar soil N_2_O fluxes compared with continuous wheat after two years. Behnke et al. [24] reported that corn emitted similar soil CO_2_ fluxes from continuous corn, soybean-corn and soybean-wheat- corn rotation. Such discrepancies suggest that the response of GHG emissions to crop rotational diversity may vary with different crop diversity, climate and soil type.

Crop rotation, especially forage crops, can offer livestock mixed grazing pastures. Grazing, which is an important component in the ICL system, is used to control weeds and pests, reduce feed costs, and increase nutrient turnover rate [25]. Grazing can impact soil GHG by modifying canopy structure and residue accumulation [26–27]. In addition, grazing alters soil temperature and water content by consuming crop residues [28–29], both of which in turn affect soil microbial processes, the primary processes responsible for soil GHG fluxes [30–32]. The material added to the soil from animal excreta deposition (dung and urine) as well as the soil compaction increased by the movement of the animals can create anaerobic environments suitable for denitrification, resulting in the acceleration of soil GHG fluxes [33–34].

Several studies in the past have reported that grazing can enhance soil GHG fluxes [35–38], however, Zhong et al. [34] found that five years of grazing did not affect N_2_O emissions compared to ungrazed plots. Other studies have reported that grazing decreased soil CO_2_ fluxes compared to ungrazed soil [39–41]. Such a reduction was attributed to various, but presently uncertain, issues that include a decrease of (1) the diffusion of CO_2_ in soil (due to the increase in soil bulk density), (2) the SOC (grazing can remove the biomass, thus reducing C input), and (3) activities of soil organisms (grazing would deplete SOC and depress microbial activities). Wolf et al. [42] reported that grazing decreased the emissions of N_2_O due to the reduction in soil organic matter and soil moisture, especially in arid and semi-arid regions. No significant difference in CH_4_ flux between grazed and ungrazed plots was reported by Wang et al. [43]. Typically, upland agricultural soils are minor emitters or minor sinks for CH_4_ [17], therefore, grazing usually has minor effects on CH_4_ flux in these systems. These results emphasize the need to explain the mechanistic reasons for differences in the impact of grazing on soil GHG fluxes.

Current knowledge cannot explain the mechanisms responsible for the crop rotational diversity and grazing effects under the ICL system on soil GHG emissions in the semi-arid region of the Northern Great Plains. The specific objectives of this study were to study the mechanisms affecting soil GHG emission from (i) different plant species within the rotation, (ii) crop rotation and mono-cropping, and (iii) grazing during the growing season.

## Materials and methods

### Site description

A field experiment to investigate the effect of crop rotation and grazing under the ICL system on soil GHG fluxes was initiated in 2011 at the Dickinson Research Extension Center in Dunn County, North Dakota (46°53’N, 102°49’W). The present study was conducted for 2 yr: 2016 and 2017 on a Vebar Series (coarse-loamy, mixed, superactive, frigid Typic Haplustolls) and on a Savage Series (fine, smectitic, frigid Vertic Argiustolls). To minimize the impact of soil on the analysis of the ICL system effects, two replicates were assigned to one soil type and the third replicate was assigned to the other soil for each treatment. This study area is representative of a typical rainfed farming system in North Dakota. The experimental site was characterized as a continental climate with moist springs (April, May, and June), relatively dry summers (August and September), and cold and snowy winters (November through March). Before the initiation of the experiment, basic soil characterization data were collected and represented in Table 1.

**Table 1:** Basic soil properties (averaged for 0–10 cm depth) at the initiation of the study.

Crop	pH	EC^a^ (dS m^-1^)	OM (%)	N (kg ha^-1^)	P (ppm)
Continuous wheat	5.43	0.33	3.37	27.24	25.70
Wheat	5.93	0.29	2.78	20.74	26.70
Pea/barley	5.60	0.38	3.83	14.23	28.30
Sunflower	6.27	0.34	3.60	18.27	31.70
Cover crops	5.43	0.27	3.30	15.13	23.00
Corn	5.55	0.26	3.31	16.23	26.32
**Analysis of variance *P* > F**					
	0.640	0.260	0.340	0.220	0.800

^a^EC, electrical conductivity; OM, organic matter; N, nitrogen; P, phosphorus.

### Crop rotation treatments

The cropping system investigated here included mono-cropping system (spring wheat [*Triticum aestivum L*.]) grown continuously for five years and a 5-yr crop rotation (spring wheat-cover crop-corn [*Zea mays* L.]-pea [*Pisum sativum* L.]/barley [*Hordeum vulgare* L.]-sunflower [*Helianthus annuus* L.]), a total of 6 main plots, with three replicates. Species of cover crops used in this study are listed in S1 Table. Every phase of the crop was present each year. The pea/barley, corn and cover crops plots were split into two areas in 2016 and 2017 (grazed and ungrazed), where grazing occurred only for these three species. Treatments were arranged in a randomized complete block in 18 uniform rectangular 1.74 ha (31.3 ha in total) plots. All crops were seeded with a John Deere 1590 no-till drill (Deere & Company, Moline, IL). For the pea/barley field pea (*Arvika*, *var*.) mix, seeding rate was 67.2 kg ha^-1^ and the forage barley (*Stockford var*.) was seeded at the rate of 44.8 kg ha^-1^ resulting in a combined 3,087,500 estimated plants ha^-1^. Crop management decisions, based on soil test results and recommendations from the NDSU Soil Testing Laboratory, indicated that no nitrogen fertilizer needed to be applied to the crops in 2016 and 2017. However, herbicides for grass and broadleaf weed control were applied, as needed for weed control. All crops were grown under rain fed condition, and no irrigation was applied. Crop managements are included in Table 2.

**Table 2:** Agronomic and grazing management information at the study site performed during 2016 and 2017.

Crop	Planting Date	Population (plants ha^–1)^	Seeding depth (cm)	Hybrid	Row spacing (cm)	Harvest date	Grazing start date	Grazing end date
**2016**								
Continuous wheat	9-May	3,087,500	2.54	Barlow	19.10	16-Aug.		
Wheat	9-May	3,087,500	2.54	Barlow	19.10	16-Aug.		
Sunflower	9-May	49,400	5.08	60ME80	76.20	9-Nov.		
Pea/barley	9-May	3,087,500	3.81	Mixed	19.10		20-July	16-Aug.
Corn	21-May	49,400	5.08	Master graze BMR	76.20		16-Aug.	5-Oct.
Cover crops	13-July	3,730,170	2.54	13 species	19.10		5-Oct.	11-Nov.
**2017**								
Continuous wheat	3-May	3,087,500	2.54	Barlow	19.10	18-Aug.		
Wheat	3-May	3,087,500	2.54	Barlow	19.10	18-Aug.		
Sunflower	15-May	49,400	5.08	60ME80	76.20	19-Oct.		
Pea/barley	1-May	3,087,500	2.54	Mixed	19.10		11-July	16-Aug.
Corn	9-May	49,400	5.08	Master graze BMR	76.20		16-Aug.	23-Sep.
Cover crops	15-June	3,730,170	2.54	13 species	19.10		23-Sep.	23-Oct.

### Grazing treatments

Spring wheat and sunflower were cash crops in the cropping system, and yearling crossbred beef steers grazed the pea/barley, corn and cover crops plots in 2016 and 2017. When grazing started, the experimental design was modified to incomplete split-plot with pea/barley, corn and cover crops plots split in two grazing treatments (grazed and ungrazed). Protocols were reviewed and approved for animal use in this investigation by the North Dakota State University Institutional Animal Care and Use Committee (Protocol Approval #A16015). Before grazing started in the experimental plots in 2016 and 2017, the yearling beef cattle steers grazed native range from May 4 to July 11 (68 d) in both years to allow adequate time for the annual forages that were to be grazed to attain sufficient growth before grazing began. Based on crop growth, grazing in both years began in the pea/barley plot first before moving to the corn and finally to the cover crops plots. Based on best grazing management practices, the harvest efficiency goal was to utilize approximately one-half or the available forage and leave one-half for soil armor, resulting in the number of hectares grazed per steer per month of 0.211, 0.143, and 0.25 for pea/barley, corn, and cover crops, respectively. Grazing information including the beginning and end of grazing are included in Table 2. The yearling steers grazed in the appropriate fields at all times from the beginning of the grazing until they were moved to the next plot.

### Soil sampling and analysis

At planting in 2016 and 2017, four samples from random spots in each plot, including grazed and ungrazed plots from 2016 grazing, were collected at 0–5 cm depth using a push probe auger (3.2-cm diam.), composited and air-dried at ambient temperature, and ground to pass through a 2-mm screen for determining SOC and total nitrogen (TN) using the method outlined by Nelson and Sommers [44]. In addition, soil bulk density from all plots at planting in 2016 and 2107 were measured from 0–5 cm depth using the core method [45]. No soil samples were collected after 2017 grazing.

### Measurements and analysis of GHG fluxes

Soil GHG fluxes from all the plots were measured for 2016 and 2017 using the static chamber technique described by Parkin and Venterea [46]. The chambers, which were made of polyvinyl chloride (PVC) pipe anchors (20.3 internal diameter and 15 cm height), were installed in each plot at the beginning of the study and remained undisturbed during the entire monitoring period except for temporary removal when mechanized farm operations were performed. A minimum of 24 h elapsed before resumption of sampling activities following chamber installation. The gas samples were collected once a week depending on weather conditions from June through October of 2016 and 2017. The GHG fluxes were measured only during the growing seasons, and the sampling was discontinued during periods when the installation of the chambers on the collars would have necessitated disturbance of the snow cover causing a non-representative sampling location (≈ four months each year). Sampling was typically done between 8:00 am and noon, with gas samplings being taken during the same period each day to reduce the diurnal effect of temperature on GHG fluxes. A PVC cap with a vent tube and sampling port were placed on anchors prior to taking the gas samples. A lid was used to keep these chambers closed during the gas sample collection. The gas samples from the chamber were collected with a syringe (10 mL) at 0, 20 and 40 minutes and transferred into 10 mL glass vials sealed with butyl rubber septa pre-charged inert argon gas that was removed by needle puncture prior to transfer of collected gas samples from the static chambers. Two chambers were installed per plot to reduce spatial variability in GHG measurement, and the average value was used for each treatment for data analysis.

Air chamber temperature during the time of sampling from each chamber was measured for calculating the GHG fluxes. The gas samples were analyzed using a gas chromatograph (CombiPAL; CTCAnalytics, Zwingen, Switzerland), which was fully automated with thermoconductivity, flame ionization, and electron capture detectors for analysis of CO_2_, CH_4_, and N_2_O concentrations in one gas sample. Daily GHG flux was calculated as:

$$F=\rho \left(\frac{V}{A}\right)\left(\frac{\Delta c}{\Delta t}\right)273/\left(273+T\right)$$ (1)

where *F* is gas flux, *ρ* is gas density under normal conditions (mg m^-3^), *V* is the volume of the static chamber (m^3^), *A* is the area that the static chamber covered, *Δc/Δt* is changes in gas concentration (*Δc*) during a certain time (*Δt*), and *T* is mean temperature inside the chamber. Cumulative fluxes for each crop growing season in a year was calculated using linear interpolation.

As a supplement to gas flux measurements, soil water content and temperature at the 0 to 5 cm depth were measured using a HH2 moisture sensor (Delta-T-Devices, Cambridge, England) and a thermometer (Acurite Digital Meat Thermometer, 00641W, AcuRite Company, Geneva, WI), respectively. Average daily minimum and maximum air temperature and precipitation (mm) for each year (2016 and 2017) were collected from a weather station located approximately 25 km from the field.

### Statistical analysis

Data were normal (Skewness and Kurtosis’s tests) and homogeneous (Levene’s test) for all the variables. To determine the effect of crop rotational diversity and grazing, data for GHG fluxes were analyzed using the GLIMMIX procedure in SAS (SAS Institute Inc., North Carolina, U.S.). Sampling date, year, soil, crop and their replicates were defined as random variables. Fixed effects in the model included crop phase ‘nested’ within grazing. Mean values were separated using pairwise differences method (adjusted by Tukey). Analysis of variance (ANOVA) was conducted to investigate the effect of crop rotational diversity and grazing on average soil temperature and soil water content. Linear (Y = B_0_+B_1_X_1_) and multiple linear regression analysis (Y = B_0_+B_1_X_1_+B_2_X_2_) were conducted to examine the relationship between soil temperature and soil water content with CO_2_ and N_2_O fluxes using SIGMA PLOT 14.0. Differences were considered significant at 0.05 probability.

## Results

### Climate, soil temperature and water content

**Fig 1:**
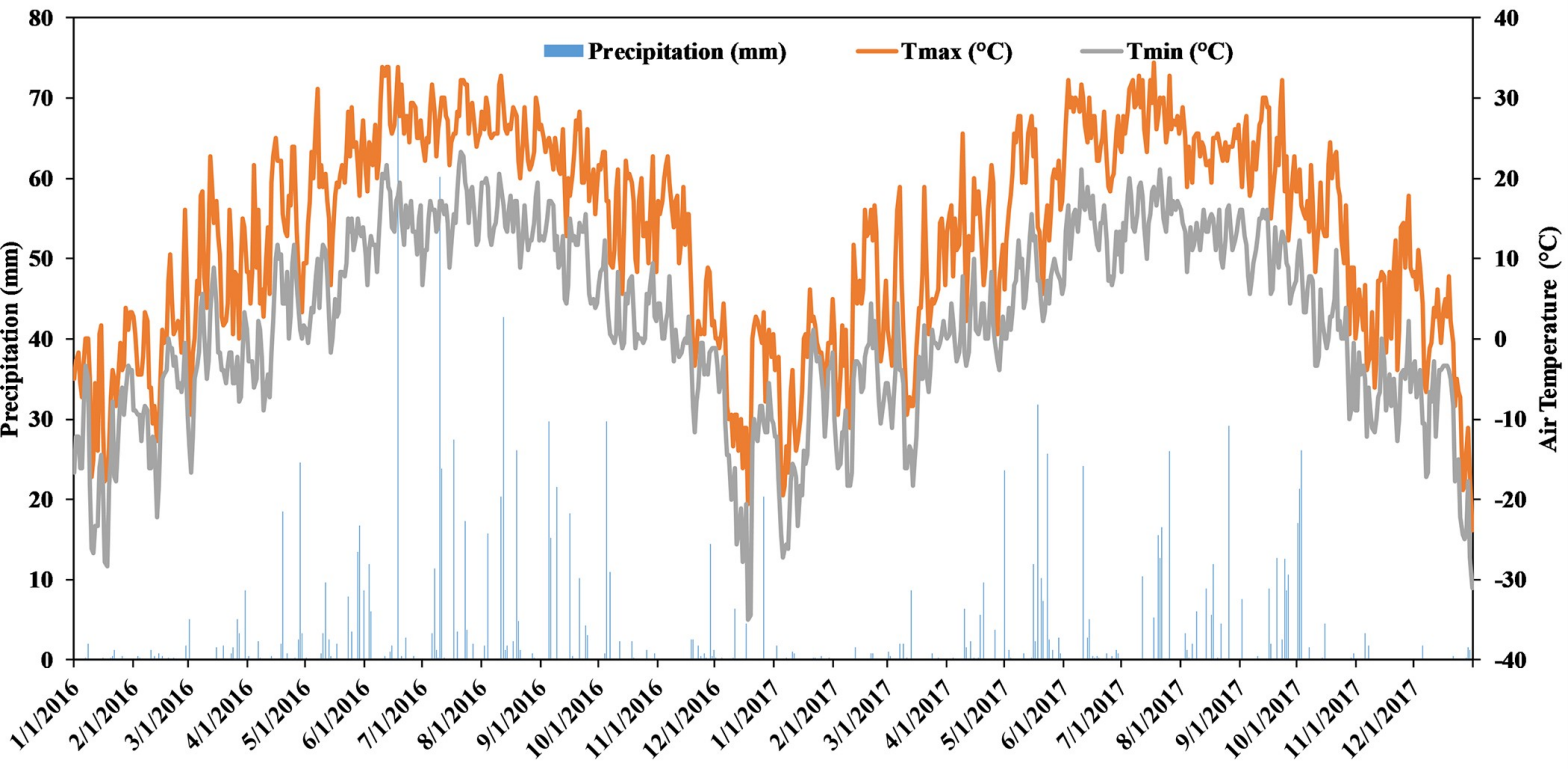
Monthly average precipitation (PRCP), air maximum (TMAX) and minimum temperature (TMIN) for the 2016 and 2017. Weather data from Agrometeorological Station located 25 km away from the experimental site.

**Fig 2:**
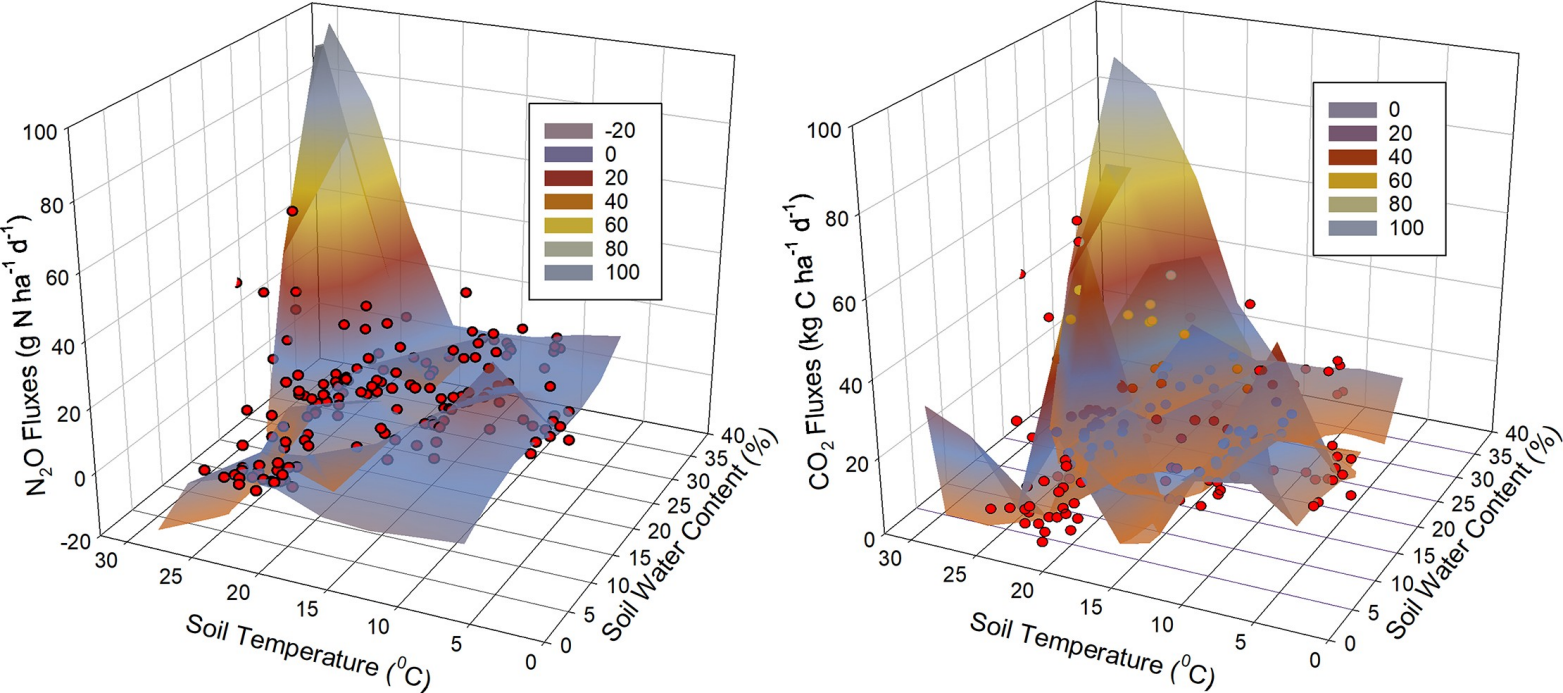
Effects of soil temperature (°C) and soil water content (%) on CO_2_ and N_2_O fluxes over the study period (2016 and 2017).

The mean values of precipitation and air temperature for 2016 and 2017 are shown in Fig 1. Average (30-yr) air temperature at the site was 6.2°C, and mean annual precipitation was 610 mm. Total precipitation in 2016 (740.7 mm) was 29.7% higher than in the 2017 (520.3 mm) (Fig 1). Generally, the spring and summer of 2016 were unusually wet with higher precipitation throughout the growing season. The precipitation during the growing season (May–October) was greater in 2016 than in 2017. Air temperature was similar during the growing season in the two years. The air temperature values at the beginning of the growing seasons for both years rose before falling later in the season (Fig 1). Soil temperature and water content (S1 and S2 Figs) were not affected by crop rotational diversity and grazing over the study period (averaged across measurements dates, *p* >0.05). The linear regressions for the CO_2_ and N_2_O fluxes and soil temperature showed a significant positive correlation between soil temperature and the CO_2_ fluxes (R^2^ = 0.63 and *p* = 0.021) and N_2_O fluxes (R^2^ = 0.71 and *p* = 0.011) over the two years of this study. However, non-significant correlations between soil water content and CO_2_ fluxes (R^2^ = 0.16 and *p* = 0.121) and N_2_O fluxes (R^2^ = 0.21 and *p* = 0.834) were found in this study. The CO_2_ and N_2_O fluxes plotted against soil temperature and water content over the study period are shown in Fig 2. The results from multiple regression analysis showed significant positive correlations between the combination of soil water content and soil temperature with CO_2_ and N_2_O fluxes (*p* <0.001 for CO_2_ fluxes and *p* = 0.0051 for N_2_O fluxes). This combination (soil temperature and water content) can explain up to 26% of the variation in CO_2_ fluxes and 64% of the variation in N_2_O fluxes. Maximum CO_2_ and N_2_O fluxes appeared generally at soil water content >26% and soil temperatures warmer than 27°C (Fig 2).

### Effect of crop rotational diversity on GHG fluxes

**Fig 3:**
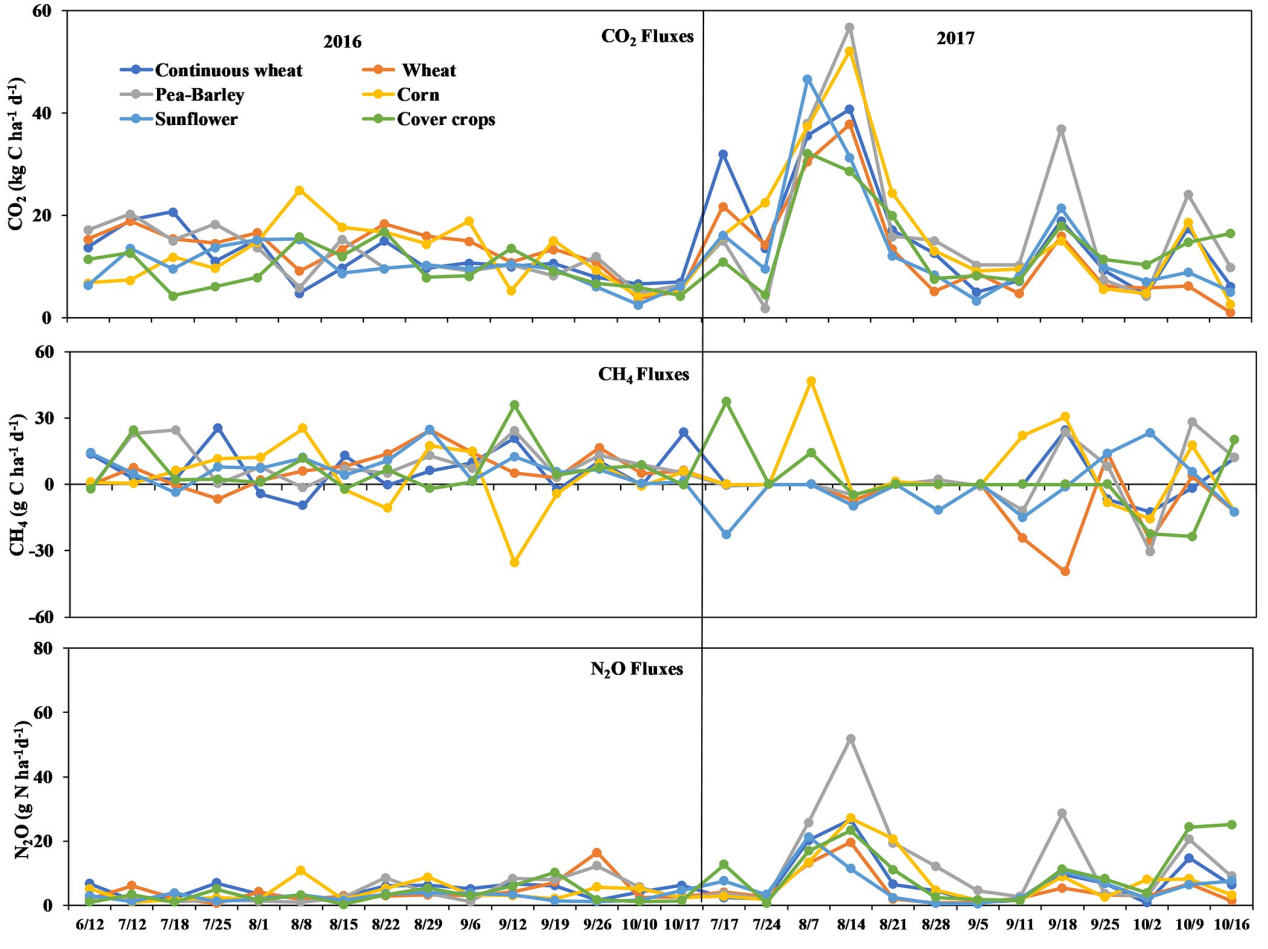
Trend of CO_2_, CH_4_ and N_2_O fluxes in the 2016 and 2017 growing seasons as influenced by crop rotational diversity.

Daily GHG fluxes based on crop phases in 2016 and 2017 are shown in Fig 3. Regardless of crop phases, soil GHG fluxes were lower during the 2016 than the 2017 season (Fig 3). Cumulative GHG fluxes from all crop phases were higher in 2017 compared to the 2016 (Table 3).

**Table 3:** Significance of F values for year for GHG cumulative fluxes.

	Nparm	DF	Sum of squares	F ratio	Prob > F
CO_2_	1	1	459272.7	2.18	0.0148
CH_4_	1	1	5550524.0	13.49	0.0008
N_2_O	1	1	1749469.2	10.53	0.0026

The trend in soil CO_2_ fluxes under all the crop phases exhibited the same pattern for all sampling dates (Fig 3). The highest peak of CO_2_ flux over the two years from crop phases was recorded on August 14, 2017 (Fig 3) under pea/barley (56.8 kg C ha^-1^ d^-1^), corn (52.1 kg C ha^-1^ d^-1^), continuous spring wheat (40.7 kg C ha^-1^ d^-1^), spring wheat (37.8 kg C ha^-1^ d^-1^), sunflower (31.3 kg C ha^-1^ d^-1^), and cover crops (28.7 kg C ha^-1^ d^-1^). No significant differences in the daily CO_2_ flux was observed among all the crop phases during this peak (*p* = 0.5341, Fig 3). For other sampling dates, no significant effect of crop rotational diversity (*p* ≥0.05 for each sampling date for each year) on CO_2_ fluxes was found. Cumulative CO_2_ fluxes exhibited no significant differences among all phases over the study period (Table 4). Comparing the effect of mono-cropping with rotation on CO_2_ flux revealed that continuous spring wheat resulted in similar cumulative CO_2_ flux compared to spring wheat over the study period (Table 4).

**Table 4:** Cumulative soil surface CO_2,_ CH_4_ and N_2_O fluxes influenced by crop rotational diversity and grazing over the study period.

		CO_2_ (kg C ha^-1^)	CH_4_ (g C ha^-1^)	N_2_O (g N ha^-1^)
**Rotation**				
Continuous wheat		1570.17a^a^	520.54a	671.46b
Wheat		1583.73a	128.18a	571.57c
Pea/barley		1889.85a	369.32a	1071.40a
Sunflower		1420.87a	416.91a	500.89c
Cover crops		1454.96a	528.74a	746.94b
Corn		1777.02a	609.42a	722.74b
**Grazing**^b^				
Pea/barley	Grazed	642.91a	-45.46a	443.81a
	Ungrazed	642.08a	53.07a	444.51a
Corn	Grazed	349.18b	214.54a	252.41a
	Ungrazed	459.17a	122.38a	187.46a
Cover crops	Grazed	85.78b	118.87a	51.38a
	Ungrazed	126.18a	-87.50a	148.80a
Average	Grazed	359.29b	95.90a	249.20a
	Ungrazed	409.14a	29.31a	260.29a

^a^Within a column, values followed by the same letters are not significantly different at a = 0.05.

^b^Cumulative GHG was only for the period when grazing occurred.

The trend of the CH_4_ fluxes under crop phases varied on the sampling dates over the two years (Fig 3). Generally, peaks of CH_4_ release were observed after rainfall events, while peaks of CH_4_ uptake corresponded with an increase in soil temperature. No significant effects were observed for all phases on the CH_4_ fluxes during these peaks and other sampling dates for both years (*p* >0.05 for all sampling dates). Although not significant, CH_4_ effluxes have negative peaks very much pronounced for corn and wheat compared to other crops. In addition, no significant effects from crop rotational diversity on cumulative CH_4_ fluxes were observed (Table 4).

Similar to the trend of the CO_2_ fluxes, the variation in N_2_O fluxes exhibited the same pattern under all phases on all sampling dates for both years (Fig 3). The highest peak of N_2_O flux over the two years was recorded on August 14, 2017, the same date as the highest peak of CO_2_ flux (Fig 3). This N_2_O peak occurred under pea/barley (51.85 g N ha^-1^ d^-1^), a peak that was significantly higher (*p* = 0.001) than for the other phases (27.1 g N ha^-1^ d^-1^ for corn, 26.7 g N ha^-1^ d^-1^ for continuous spring wheat, 19.6 g N ha^-1^ d^-1^ for spring wheat, 11.3 g N ha^-1^ d^-1^ for sunflower, and 23.2 g N ha^-1^ d^-1^ for cover crops). Except for the peak in N_2_O fluxes, no significant effect of crop rotational diversity (*p* ≥0.05 for each sampling date) on N_2_O fluxes for both years was found. N_2_O fluxes were higher in continuous spring wheat compared to spring wheat for 19 of the 29 sampling dates over the 2016 and 2017 period; however, no significant differences in N_2_O fluxes between these two phases were observed (*p* ≥0.05 for each sampling date). Crop rotational diversity over the study period affected the cumulative N_2_O fluxes, with pea/barley recording higher cumulative N_2_O fluxes than the other phases (Table 4). Comparing the effect of mono-cropping with crop rotation on cumulative N_2_O fluxes found that continuous spring wheat resulted in greater cumulative N_2_O fluxes compared to spring wheat (Table 4).

### Effect of grazing on GHG fluxes

**Fig 4:**
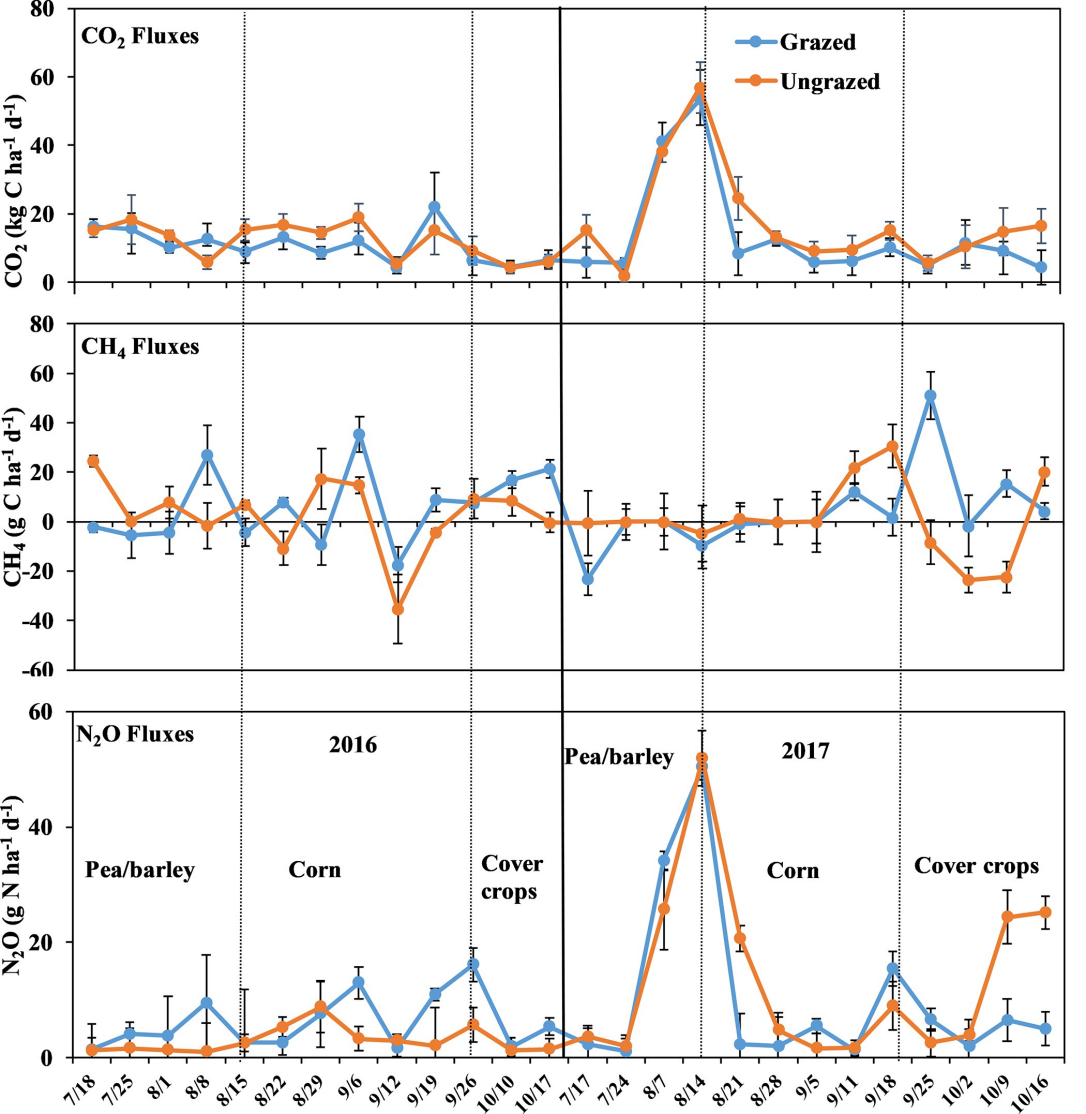
Trend of CO_2_, CH_4_ and N_2_O fluxes as influenced by grazing during the grazing period of pea/parley, corn and cover crops for the 2016 and 2017 growing seasons. Vertical bars indicate standard errors.

The GHG fluxes based on grazing in 2016 and 2017 are shown in Fig 4. Cumulative GHG fluxes from grazing are listed in Table 4. Similar to the trend in daily soil CO_2_ fluxes under crop phases in rotation, the trend in daily soil CO_2_ fluxes under grazing treatments appeared to be similar on all sampling dates (Fig 4). On August 14, 2017, the same date when the highest peak was recoded under crop phases in rotation, the highest peak of CO_2_ flux over the two years was recorded for the pea/barley plots (Figs 3 and 4). No significant differences (*p* = 0.543) on the daily CO_2_ fluxes were recorded during this peak (53.3 kg C ha^-1^ d^-1^ for grazed plots and 56.6 kg C ha^-1^ d^-1^ for ungrazed plots, Fig 4). For other sampling dates, no significant effect of grazing (*p* ≥0.05 for each sampling date for each year) on CO_2_ fluxes was found. Regardless of the grazed crop, cumulative CO_2_ flux was significantly lower under grazed plots than under ungrazed plots (Table 4). The trend and the peaks of the CH_4_ fluxes under grazing treatments over the two years (Fig 4) were similar to the trend and the peaks of the CH_4_ fluxes under crop phases in rotation (Fig 4). No significant effects were observed for grazing on the cumulative CH_4_ fluxes (Table 4).

The highest peak of N_2_O flux over the two years was recorded on August 14, 2017 from the pea/barley plots (Fig 4), with ungrazed plots emitting similar N_2_O flux (51.9 g N ha^-1^ d^-1^) compared to the grazed plots (50.3 g N ha^-1^ d^-1^). For other sampling dates, no significant effect of grazing (*p* ≥0.05 for each sampling date for each year) on N_2_O fluxes was observed. Grazing did not affect cumulative N_2_O flux as seen in Table 4.

## Discussion

### Soil temperature and water content

While the daily trend of soil temperature was similar to that of the maximum and minimum air temperature under crop phases in rotation and grazing during the growing season for both years, the daily trend of soil water content was highly influenced by precipitation events and varied with the measurement dates. Soil temperature and water content were not affected by crop rotational diversity over the study period. Soil organic carbon for mono-cropping and rotation indicated that both practices have similar SOC, perhaps explaining why continuous spring wheat recorded similar soil temperature and soil moisture compared to spring wheat in this reported study. Significant changes in SOC may need long-term study to be detected. King and Blesh [10] reported that enhancing SOC can improve soil water content and temperature. The continuous cropping of a similar crop leads to retarded plant growth, serious pest and disease damage, and low crop productivity [47], therefore, it was expected that continuous spring wheat would record lower SOC compared to spring wheat, however, both had similar SOC. Long-term crop rotation study (>5 years) might show the negative effects of mono-cropping on plant yield. Fu et al. [48], who investigated the 30-yr effect of crop rotation (alfalfa–potato and winter wheat) compared with mono-cropping (continuous winter wheat), reported that wheat in rotation increased SOC compared to continuous wheat in the semiarid climate. This difference in the effect of crop rotation on SOC between our study and Fu et al. [48] can be attributed to the differences in crops used in the rotation and the length of the rotation study.

Soil temperature and water content were not affected by grazing. Grazing can alter soil temperature by increasing the radiant energy reaching it, leading to higher soil temperature. Risch et al. [49] also reported that grazing grass for five years did not affect the temperature of the soil (Mollisols, like the studied soil) compared to ungrazed plots in Wyoming. In addition, soil water content was not affected by grazing, confirming the results found by Barsotti et al. [25], who conducted a study in Montana (dryland cropping systems) on silt loam soil, reported that grazing spring wheat, pea/barley hay, and alfalfa for 11 years did not affect soil water content compared to ungrazed soil. Conversely, soil water content in an ungrazed pasture was higher than in a grazed one (grazing for more than13 years) in other studies [50–51], which they attributed to the greater accumulation of litter in the ungrazed plots decreasing evaporation and thus, increasing soil water content in these plots.

### Effect of crop rotational diversity on GHG fluxes

Soil GHG fluxes under crop phases were lower during the 2016 season than the 2017 season, a result partially attributed to the higher soil temperature in 2017. Higher soil temperature can stimulate microbial activity and C and N mineralization, causing an increase in GHG fluxes [52–53,16]. The positive correlation between soil temperature and the CO_2_ and N_2_O fluxes over the two years emphasizes the important role of this parameter in their productions. Higher temperature can enhance the microbial activity needed to breakdown the organic matter and hence increase CO_2_ production. The emission of N_2_O from soils is primarily caused by microbial nitrification and denitrification, both of which are controlled by temperature: more specifically, the lower the temperature, the lower the soil microbial activity, resulting in reduced N_2_O emissions [17]. Our previous study [54] conducted in South Dakota on fine silty soil reported that GHG emissions increased with the increase in soil temperature due to the increase in the microbial activity and C and N mineralization, causing an increase in GHG emissions, consistent with our results.

The variation in soil CO_2_ fluxes under all crop phases appeared to be related to the trends in soil temperature or water content for both years. The peak of CO_2_ that observed in 2017 and not in 2016 was a response to an increase in soil temperature after precipitation events. Soil CO_2_ from root respiration is a primary contributor to total soil respiration rates in most soils, it is likely that higher soil temperature increased root respiration and microbial activities which increased CO_2_ emission in this year compared to 2016. These results were supported by Lee et al. [55], who reported that a higher soil temperature corresponded with a higher soil respiration rate due to the increase of the biological process. However, no significant effects of crop rotational diversity on the daily CO_2_ fluxes for each sampling date for each year in this study were found. In addition, no significant differences between all phases on cumulative CO_2_ fluxes were found, results that may be due to the absence effect of crop rotational diversity on soil temperature over the study period. However, Carvalho et al [56], Rochette and Janzen [57] and Brock et al [58] reported that the decomposition of the previous crop residues in the crop rotation can influence GHG fluxes. Other parameter that could influence soil respiration included SOC; however, there was no significant effect on SOC by crop rotational diversity.

The variation in daily CH_4_ fluxes under crop phases were not similar over the two years, varying both positively (atmospheric source) and negatively (atmospheric sink). Generally, peaks of CH_4_ release were observed after rainfall events, while peaks of uptake corresponded with an increase in soil temperature. Ozlu and Kumar [54] and Lee et al. [55] reported that whether a soil is a source or a sink for CH_4_ depends on the activity of the CH_4_ release microorganisms (increases under anaerobic conditions) and the CH_4_ uptake microorganisms (increases under aerobic conditions). Although not significant, CH_4_ effluxes have negative peaks very much pronounced for corn and wheat compared to other crops. The reasons for higher soil CH_4_ uptake for corn and wheat than in other phases in this study were not known. No significant effects of crop rotational diversity on daily and cumulative CH_4_ fluxes were observed in this study, perhaps because of the non-significant effect of crop rotational diversity on soil water content. Similarly, Behnke et al. [20] conducted an experiment in Northwestern Illinois (cropping system) reported that a corn-soybean rotation had similar CH_4_ fluxes from silty clay loam soil compared to continuous corn or continuous soybean systems alone after 17 years of rotation. In addition, crop rotational diversity did not affect soil bulk density (data not shown), which may explain the lack of effect from crop rotational diversity on CH_4_ fluxes in this study. In well-drained mineral soils, diffusion of CH_4_ flux into the soil is the main factor limiting the CH_4_ flux. Any changes in soil aeration can significantly affect the CH_4_ flux.

Similar to the trend of the daily CO_2_ fluxes, the variation in N_2_O fluxes showed the same pattern under crop phases on all sampling dates for both years. The highest peak of N_2_O flux was attributed to the increase in soil temperature that occurred after heavy rainfall [59]. The N_2_O flux was correlated with soil temperature and water content, suggesting N mineralization, and nitrification/denitrification processes may occur simultaneously, resulting in greater N_2_O flux. A significant effect during this peak was observed, with pea/barley recording higher N_2_O flux than the other phases. Legumes can increase N_2_O emission through atmospheric N_2_ fixation by the rhizobia living in the root nodules. This fixed N_2_ can be mineralized to release inorganic N producing N_2_O when nitrified [60–62]. However, no significant effect of crop rotational diversity on other daily N_2_O fluxes was found. Although daily N_2_O fluxes were higher in continuous spring wheat compared to spring wheat for many days during 2016 and 2017, no significant differences in daily N_2_O fluxes between these two phases were observed. Unlike cumulative CO_2_ fluxes, pea/barley recorded higher cumulative N_2_O flux than other phases, perhaps due to the ability of pea/barley to fix N_2_, increasing N_2_O emissions compared to other phases as mentioned earlier. Leguminous crops can be a source of N_2_O emissions during residue decomposition because of their greater N concentration than non-leguminous crops. The fact that leguminous plots had higher TN than other phases may explain the significant increase in cumulative N_2_O fluxes from leguminous plots compared to other plots. Sainju et al. [9] reported increased accumulation of total organic C from legumes compared to non-legumes. Comparing the effect of mono-cropping with crop rotation, continuous spring wheat resulted in greater cumulative N_2_O fluxes compared to spring wheat. Soil grown with continuous corn emitted higher N_2_O emission than soil grown with corn in rotation [30, 24]. Other soil parameters (like soil aggregates and enzyme activities) that affect GHG flux were not measured in this study. These properties may have been improved by crop rotation compared to mono-cropping as suggested by [63, 12], which can decrease soil N_2_O emissions.

### Effect of grazing on GHG fluxes

The trend of soil CO_2_ fluxes under grazing treatments (grazed and ungrazed) appeared to be related to trends in soil temperature or water content for both years as the CO_2_ peaks under grazing were in response to an increase in the soil temperature that occurred after precipitation events. Wegner et al. [17], who conducted a study on fine silty soil in South Dakota from 2013 to 2015, reported that higher soil temperature can cause an increase in CO_2_ fluxes due to the increase in microbial activity, C mineralization and root respiration, causing an increase in CO_2_ production. No significant effects of grazing on the daily CO_2_ fluxes for each sampling date for each year in this study were found. However, grazed plots recorded lower cumulative CO_2_ fluxes than ungrazed plots. Cumulative CO_2_ flux was reduced by grazing as a result of reduced C input since this biomass was removed by the grazing event, consistent with SOC being lower in grazed plot in 2016 (*p* = 0.0210). Cao et al. [64] reported that seasonal CO_2_ flux was significantly higher in a low grazed site (grazing for 13 years) in China than in a high grazed one, a difference they attributed to the lower SOC in the high grazed site. Tang et al. [40] reported that heavy-grazing for ten years decreased soil CO_2_ flux during spring-thaw period in desert steppe in China compared to ungrazed soils. It is possible that grazing in this study reduced the diffusion of CO_2_ fluxes in soil, resulting in lower cumulative CO_2_ fluxes from grazed plots compared to the ungrazed plots. The fact that grazed plots had higher soil bulk density than ungrazed plots in 2016 (*p* = 0.002) supports this conclusion. On the other hand, grazing did not affect daily and cumulative CH_4_, perhaps because of the similar soil water content between the grazing treatments. Paz-Ferreiro et al. [65] reported that sheep grazing for 14 years in Northern England (temperate weather) had no effect on CH_4_ fluxes compared to ungrazed site.

The variation in N_2_O fluxes under grazing treatments showed the same pattern on all sampling dates for both years. Denef et al. [66] and Sainju et al. [16] reported that yearling steers feces and urine returned to the soil from grazing can enhance microbial activity and N mineralization. However, grazed plots emitted similar cumulative N_2_O flux compared to ungrazed plots in this reported study. This result can be attributed to the lack effect from grazing on soil TN in 2016 (*p* = 0.341). Shaaban et al. [62] reported that soil TN content is critical in controlling soil N_2_O emissions. On the other hand, Wolf et al. [42] reported that grazing decreased annual N_2_O fluxes compared to ungrazed plots, which was attributed to the increase in the soil water content in the ungrazed plots, stimulating denitrifying microbial activity, causing the increase in the N_2_O fluxes compared to grazed plots in this cited study.

Because only three crops were grazed in this reported study, these findings are not applicable to all other plant species within the rotation. In addition, spring wheat in mono-cropping and rotation systems was used as a cash crop in this study. Therefore, comparing the effects of grazing a crop grown in rotation with a similar crop grown in mono-cropping on GHG fluxes was not evaluated. More research is required to evaluate the response of GHG emissions to grazing with different crops and soil types under different climatic conditions.

### Limitations of the study

Very limited studies are conducted across the world to evaluate the impacts of ICL system on soil surface GHG emissions. Nonetheless, like many other studies, this study had two limitations. First, the GHG fluxes were measured only during the growing season; therefore, the effect of ICL system on GHG emission is not quite conclusive. Measurements of GHG emissions over the entire year may be required to evaluate the effect of ICL system on GHG emissions. Second, this study did not include the emissions from the livestock (enteric), which can be a critical component in addressing the overall goal of the ICL system. Despite these limitations, however, we believe that this study provides important observational data assessing the effects of ICL system on soil GHG emissions.

## Conclusions

This study was conducted to assess the impact of crop rotational diversity and grazing under an ICL system on soil surface GHG emissions, and to compare the effects of crop rotation and mono-cropping on soil GHG emissions. Differences in weather conditions between the two studied years influenced soil temperature, which, in turn, affected GHG emissions. The findings indicated that crop rotational diversity did not affect cumulative CO_2_ and CH_4_ emissions. Mono-cropping (continuous spring wheat) recorded higher cumulative N_2_O fluxes than crop rotation (spring wheat). Grazing decreased cumulative CO_2_ flux compared to ungrazed, however, cumulative N_2_O and CH_4_ fluxes were not affected by grazing. Further, long-term monitoring of GHG fluxes from plots under crop rotation and grazing with different crops under different climatic conditions is required to explore sustainable strategies for mitigating the agricultural emissions.

## Supporting information

S1 Table Species of cover crops used in this study for 2016 and 2017. (PDF)

S1 Fig Soil temperature and water content as influenced by crop rotational diversity for the 2016 and 2017 growing seasons. (PDF)

S2 Fig Soil temperature and water content as influenced by grazing during the grazing period for 2016 and 2017. (PDF)

S1 File Daily means for greenhouse gas fluxes in 2016 and 2017 under different crop rotational diversity. (PDF)

S2 File Daily means for greenhouse gas fluxes in 2016 and 2017 under grazing. (PDF)
