# Supplementary material for: Impacts of crop rotational diversity and grazing under integrated crop-livestock system on soil surface greenhouse gas fluxes
Source: PLoS One. 2019 May 22;14(5):e0217069. doi: 10.1371/journal.pone.0217069 (PMC6530893; doi:10.1371/journal.pone.0217069)
Supplement: S1 Table — (PDF) [file pone.0217069.s001.pdf]

S1 Table. Species of cover crops used in this study for 2016 and 2017.

**Crop**	**% of Mix**
field pea (*Pisum sativum* L.)	46.2
stockford barley [*Hordeum vulgare* L.]	11.66
everleaf oat [*Avena sativa*]	11.66
indianhead lentil [*Lens culinaris*]	6.99
german millet [*Pennisetum glaucum*]	4.66
sorghum sudan [*Sorghum bicolor*]	4.66
Sunflower [*Helianthus annuus L.*]	4.66
bayou kale [*Brassica oleracea var. sabellica*]	2.33
dwarf essex rape [*Brassica napus*]	2.33
purple top turnip [*Brassica rapa subsp. rapa*]	2.33
berseem clover [*Trifolium alexandrinum*]	0.70
crimson clover [*Trifolium incarnatum*]	0.70
red clover [*Trifolium pratense*]	0.70
