# Supplementary material for: Impacts of crop rotational diversity and grazing under integrated crop-livestock system on soil surface greenhouse gas fluxes
Source: PLoS One. 2019 May 22;14(5):e0217069. doi: 10.1371/journal.pone.0217069 (PMC6530893; doi:10.1371/journal.pone.0217069)
Supplement: S2 Fig — (PDF) [file pone.0217069.s003.pdf]

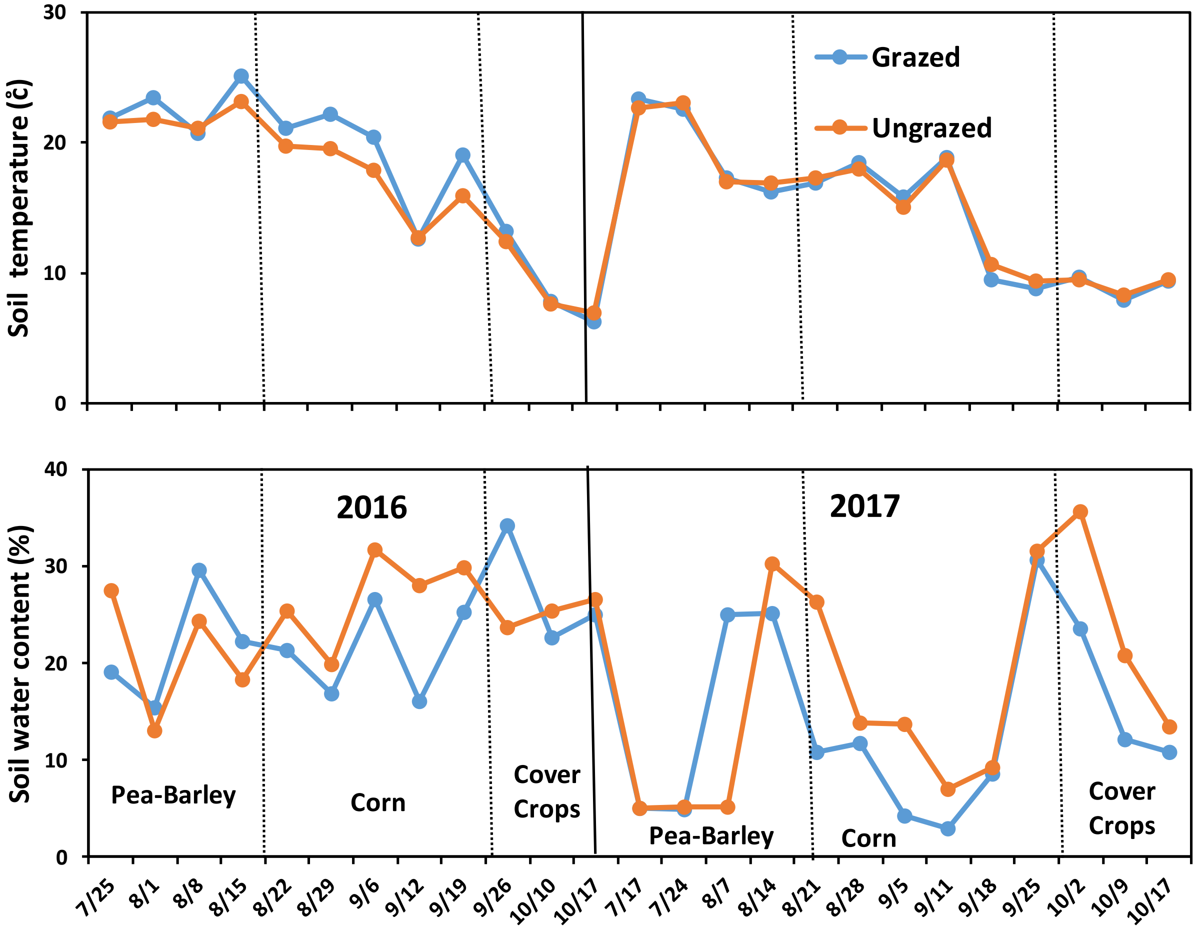

S2 Fig. Soil temperature and water content as influenced by grazing during the grazing period for 2016 and 2017.
