# Supplementary material for: Impacts of crop rotational diversity and grazing under integrated crop-livestock system on soil surface greenhouse gas fluxes
Source: PLoS One. 2019 May 22;14(5):e0217069. doi: 10.1371/journal.pone.0217069 (PMC6530893; doi:10.1371/journal.pone.0217069)
Supplement: S1 File — (PDF) [file pone.0217069.s004.pdf]

Daily means for CO_2 (kg ha^{-1} d^{-1}) in 2016 and 2017 under different crop different crop rotational diversity

GHG	Year	Date	Continuous wheat	Wheat	Pea/Barley	Corn	Sunflower	Cover crops
CO2	2016	6/12	13.77	15.38	17.17	6.83	6.39	11.42
CO2	2016	7/12	19.11	18.92	20.27	7.37	13.59	12.72
CO2	2016	7/18	20.70	15.39	15.12	11.87	9.57	4.28
CO2	2016	7/25	11.04	14.59	18.29	9.71	13.78	6.08
CO2	2016	8/1	15.36	16.61	13.68	14.82	15.20	7.86
CO2	2016	8/8	4.83	9.18	5.87	24.97	15.39	15.84
CO2	2016	8/15	9.69	13.34	15.36	17.69	8.70	12.00
CO2	2016	8/22	15.04	18.35	9.61	16.74	9.66	16.81
CO2	2016	8/29	9.71	15.93	10.27	14.43	10.30	7.87
CO2	2016	9/6	10.71	14.97	9.14	18.92	9.47	8.17
CO2	2016	9/12	10.03	10.84	10.39	5.27	10.47	13.53
CO2	2016	9/19	10.65	13.39	8.22	15.11	9.58	9.06
CO2	2016	9/26	7.92	10.88	11.98	9.28	6.10	6.68
CO2	2016	10/10	6.63	3.81	4.91	4.19	2.58	5.95
CO2	2016	10/17	6.97	5.10	6.30	5.47	6.19	4.31
CO2	2017	7/17	32.01	21.76	15.12	16.02	16.13	10.99
CO2	2017	7/24	13.57	14.28	1.92	22.52	9.59	4.50
CO2	2017	8/7	35.61	30.59	38.00	37.45	46.72	32.15
CO2	2017	8/14	40.79	37.85	56.85	52.16	31.36	28.65
CO2	2017	8/21	17.16	13.42	15.89	24.45	12.10	20.01
CO2	2017	8/28	12.64	5.19	15.12	13.08	8.42	7.60
CO2	2017	9/5	5.04	8.75	10.29	9.10	3.34	8.22
CO2	2017	9/11	7.24	4.74	10.26	9.52	8.15	7.31
CO2	2017	9/18	18.97	15.99	36.89	15.11	21.45	18.03
CO2	2017	9/25	9.26	6.24	7.41	5.60	9.98	11.42
CO2	2017	10/2	4.48	5.84	4.30	4.78	7.12	10.33
CO2	2017	10/9	17.50	6.23	24.11	18.63	8.88	14.78
CO2	2017	10/16	6.15	1.02	9.84	2.66	5.09	16.47

Daily means for CH4 (g ha^{-1} d^{-1}) in 2016 and 2017 under different crop different crop rotational diversity

GHG	Year	Date	Continuous wheat	Wheat	Pea/Barley	Corn	Sunflower	Cover crops
CH4	2016	6/12	13.54	-0.27	-1.46	0.89	14.09	-2.22
CH4	2016	7/12	2.59	7.46	23.08	0.35	4.77	24.54
CH4	2016	7/18	2.44	-0.36	24.52	6.08	-3.75	2.03
CH4	2016	7/25	25.40	-6.68	0.29	11.59	7.89	2.32
CH4	2016	8/1	-4.36	1.78	7.75	12.10	7.23	0.82
CH4	2016	8/8	-9.64	5.98	-1.64	25.24	11.94	11.70
CH4	2016	8/15	12.89	8.14	6.94	-2.47	4.08	-2.11
CH4	2016	8/22	-0.40	13.65	4.99	-10.84	10.80	6.80
CH4	2016	8/29	6.09	24.44	12.92	17.32	24.60	-1.84
CH4	2016	9/6	9.85	14.51	7.21	14.75	1.63	1.14
CH4	2016	9/12	20.78	5.03	24.22	-35.39	12.38	35.78
CH4	2016	9/19	-2.54	2.98	3.16	-4.16	5.44	4.27
CH4	2016	9/26	10.22	16.50	13.13	9.27	6.69	7.33
CH4	2016	10/10	-0.52	4.83	8.72	-0.82	0.32	8.54
CH4	2016	10/17	23.43	6.14	5.12	5.76	1.15	-0.37
CH4	2017	7/17	0.00	0.00	-0.62	0.00	-22.86	37.35
CH4	2017	7/24	0.00	0.00	0.00	0.00	0.00	0.00
CH4	2017	8/7	-0.09	0.00	0.00	46.67	0.00	14.34
CH4	2017	8/14	-9.73	-7.36	-4.76	-5.14	-9.78	-4.77
CH4	2017	8/21	0.00	0.00	0.00	1.16	0.00	0.00
CH4	2017	8/28	0.00	0.00	2.19	-0.21	-11.79	0.00
CH4	2017	9/5	0.00	0.00	-0.78	0.00	0.16	0.00
CH4	2017	9/11	0.00	-24.48	-12.00	22.03	-14.95	0.00
CH4	2017	9/18	24.36	-39.55	23.58	30.56	-1.22	0.00
CH4	2017	9/25	-6.88	13.65	8.07	-8.41	13.99	0.00
CH4	2017	10/2	-12.51	-25.42	-30.37	-15.69	23.23	-22.45
CH4	2017	10/9	-1.79	3.62	28.26	17.60	5.67	-23.62
CH4	2017	10/16	12.04	-12.58	12.04	-12.58	-12.58	20.23

Daily means for N2O (g ha^{-1} d^{-1}) in 2016 and 2017 under different crop different crop rotational diversity

GHG	Year	Date	Continuous wheat	Wheat	Pea/Barley	Corn	Sunflower	Cover crops
N2O	2016	6/12	6.79	2.12	2.31	5.19	3.07	1.02
N2O	2016	7/12	1.61	6.11	2.27	1.01	1.27	3.33
N2O	2016	7/18	2.24	2.22	1.27	1.86	3.96	1.04
N2O	2016	7/25	6.96	0.66	1.63	2.42	1.30	5.04
N2O	2016	8/1	3.57	4.24	1.35	1.88	2.05	1.71
N2O	2016	8/8	2.02	1.73	1.01	10.78	3.28	3.27
N2O	2016	8/15	2.78	2.91	2.52	1.88	1.57	0.26
N2O	2016	8/22	6.04	2.93	8.55	5.32	3.57	3.31
N2O	2016	8/29	6.19	3.32	3.66	8.78	3.91	5.36
N2O	2016	9/6	5.21	3.06	1.17	3.31	3.80	2.92
N2O	2016	9/12	6.66	4.27	8.34	3.01	3.41	5.99
N2O	2016	9/19	6.17	7.18	8.05	2.05	1.45	10.22
N2O	2016	9/26	1.58	16.40	12.31	5.70	1.27	1.79
N2O	2016	10/10	4.11	2.46	5.68	5.31	1.63	1.29
N2O	2016	10/17	6.21	2.72	2.28	2.46	4.63	1.46
N2O	2017	7/17	2.46	4.07	3.67	2.81	7.60	12.71
N2O	2017	7/24	1.94	2.63	1.97	1.91	3.39	0.72
N2O	2017	8/7	20.19	13.18	25.71	13.49	21.30	16.97
N2O	2017	8/14	26.76	19.65	51.86	27.17	11.39	23.29
N2O	2017	8/21	6.54	1.95	19.40	20.65	2.37	11.12
N2O	2017	8/28	4.59	0.72	12.15	4.79	0.59	2.48
N2O	2017	9/5	0.90	0.99	4.67	1.62	0.52	1.73
N2O	2017	9/11	1.54	2.28	2.71	1.68	2.21	1.60
N2O	2017	9/18	9.65	5.35	28.62	8.98	10.69	11.28
N2O	2017	9/25	6.84	3.31	3.66	2.56	6.82	8.21
N2O	2017	10/2	0.79	2.88	2.63	8.07	2.32	3.87
N2O	2017	10/9	14.72	6.63	20.59	8.25	6.50	24.36
N2O	2017	10/16	6.38	1.42	9.15	3.22	7.60	25.14
