# Supplementary material for: Impacts of crop rotational diversity and grazing under integrated crop-livestock system on soil surface greenhouse gas fluxes
Source: PLoS One. 2019 May 22;14(5):e0217069. doi: 10.1371/journal.pone.0217069 (PMC6530893; doi:10.1371/journal.pone.0217069)
Supplement: S2 File — (PDF) [file pone.0217069.s005.pdf]

Daily means for CO_2 (kg ha^{-1} d^{-1}) in 2016 and 2017 under different grazing treatments (grazed and ungrazed)

GHG	Crop	Year	Date	Grazed	Ungrazed
CO2	Pea-barley	2016	7/18	16.22	15.12
CO2	Pea-barley	2016	7/25	15.60	18.29
CO2	Pea-barley	2016	8/1	10.13	13.68
CO2	Pea-barley	2016	8/8	12.63	5.87
CO2	Pea-barley	2016	8/15	8.91	15.36
CO2	Corn	2016	8/22	13.09	16.74
CO2	Corn	2016	8/29	8.56	14.43
CO2	Corn	2016	9/6	12.05	18.92
CO2	Corn	2016	9/12	4.43	5.27
CO2	Corn	2016	9/19	21.93	15.11
CO2	Corn	2016	9/26	6.43	9.28
CO2	Cover crops	2016	10/10	4.44	4.31
CO2	Cover crops	2016	10/17	6.38	5.95
CO2	Pea-barley	2017	7/17	5.92	15.12
CO2	Pea-barley	2017	7/24	5.69	1.92
CO2	Pea-barley	2017	8/7	41.01	38.00
CO2	Pea-barley	2017	8/14	53.37	56.85
CO2	Corn	2017	8/21	8.46	24.45
CO2	Corn	2017	8/28	12.56	13.08
CO2	Corn	2017	9/5	5.77	9.10
CO2	Corn	2017	9/11	6.14	9.52
CO2	Corn	2017	9/18	10.14	15.11
CO2	Corn	2017	9/25	4.93	5.60
CO2	Cover crops	2017	10/2	11.38	10.33
CO2	Cover crops	2017	10/9	9.24	14.78
CO2	Cover crops	2017	10/16	4.37	16.47

Daily means for CH_4 (g ha^{-1} d^{-1}) in 2016 and 2017 under different grazing treatments (grazed and ungrazed)

GHG	Crop	Year	Date	Grazed	Ungrazed
CH4	Pea-barley	2016	7/18	-2.12	24.52
CH4	Pea-barley	2016	7/25	-5.46	0.29
CH4	Pea-barley	2016	8/1	-4.44	7.75
CH4	Pea-barley	2016	8/8	26.97	-1.64
CH4	Pea-barley	2016	8/15	-4.41	6.94
CH4	Corn	2016	8/22	7.91	-10.84
CH4	Corn	2016	8/29	-9.36	17.32
CH4	Corn	2016	9/6	35.33	14.75
CH4	Corn	2016	9/12	-17.45	-35.39
CH4	Corn	2016	9/19	8.86	-4.16
CH4	Corn	2016	9/26	7.59	9.27
CH4	Cover crops	2016	10/10	16.90	8.54
CH4	Cover crops	2016	10/17	21.42	-0.37
CH4	Pea-barley	2017	7/17	-23.32	-0.62
CH4	Pea-barley	2017	7/24	0.00	0.00
CH4	Pea-barley	2017	8/7	0.00	0.00
CH4	Pea-barley	2017	8/14	-9.60	-4.76
CH4	Corn	2017	8/21	-0.98	1.16
CH4	Corn	2017	8/28	-0.26	-0.21
CH4	Corn	2017	9/5	0.00	0.00
CH4	Corn	2017	9/11	11.93	22.03
CH4	Corn	2017	9/18	1.71	30.56
CH4	Corn	2017	9/25	51.02	-8.41
CH4	Cover crops	2017	10/2	-1.69	-23.62
CH4	Cover crops	2017	10/9	15.40	-22.45
CH4	Cover crops	2017	10/16	4.21	20.23

Daily means for N_2O (g ha^{-1} d^{-1}) in 2016 and 2017 under different grazing treatments (grazed and ungrazed)

GHG	Crop	Year	Date	Grazed	Ungrazed
N2O	Pea-barley	2016	7/18	1.39	1.27
N2O	Pea-barley	2016	7/25	4.07	1.63
N2O	Pea-barley	2016	8/1	3.81	1.35
N2O	Pea-barley	2016	8/8	9.47	1.01
N2O	Pea-barley	2016	8/15	2.59	2.52
N2O	Corn	2016	8/22	2.62	5.32
N2O	Corn	2016	8/29	7.58	8.78
N2O	Corn	2016	9/6	12.97	3.31
N2O	Corn	2016	9/12	1.62	3.01
N2O	Corn	2016	9/19	10.95	2.05
N2O	Corn	2016	9/26	16.10	5.70
N2O	Cover crops	2016	10/10	1.96	1.29
N2O	Cover crops	2016	10/17	5.40	1.46
N2O	Pea-barley	2017	7/17	2.34	3.67
N2O	Pea-barley	2017	7/24	1.11	1.97
N2O	Pea-barley	2017	8/7	34.16	25.71
N2O	Pea-barley	2017	8/14	50.37	51.86
N2O	Corn	2017	8/21	2.27	20.65
N2O	Corn	2017	8/28	1.98	4.79
N2O	Corn	2017	9/5	5.49	1.62
N2O	Corn	2017	9/11	1.35	1.68
N2O	Corn	2017	9/18	15.43	8.98
N2O	Corn	2017	9/25	6.56	2.56
N2O	Cover crops	2017	10/2	1.98	3.87
N2O	Cover crops	2017	10/9	6.50	24.36
N2O	Cover crops	2017	10/16	4.99	25.14
